# Focal adhesion kinase promotes ribosome biogenesis to drive advanced thyroid cancer cell growth and survival

**DOI:** 10.3389/fonc.2025.1252544

**Published:** 2025-05-19

**Authors:** Meghan D. Kellett, Vibha Sharma, Madeline E. Sherlock, Umarani Pugazhenthi, Madison M. Rose, Molishree U. Joshi, Monika Dzieciatkowska, Vu Nguyen, Philip Reigan, Kirk C. Hansen, Jeffrey S. Kieft, Rebecca E. Schweppe

**Affiliations:** ^1^ Division of Endocrinology, Metabolism, and Diabetes, Department of Medicine, University of Colorado, Aurora, CO, United States; ^2^ Department of Biochemistry and Molecular Genetics, University of Colorado, Aurora, CO, United States; ^3^ Department of Pharmacology, University of Colorado, Aurora, CO, United States; ^4^ University of Colorado Cancer Center, University of Colorado, Aurora, CO, United States; ^5^ Division of Structural Biology and Biophysics, Department of Medicine, University of Colorado, Aurora, CO, United States; ^6^ Department of Pharmaceutical Sciences, Skaggs School of Pharmacy and Pharmaceutical Sciences, University of Colorado, Aurora, CO, United States

**Keywords:** thyroid cancer, focal adhesion kinase, nucleolus, ribosomal biogenesis, NOP56

## Abstract

**Introduction:**

Advanced thyroid cancer, including papillary (PTC) and anaplastic thyroid cancer (ATC), are the leading causes of endocrine cancer deaths. Thus, there is a critical need to identify novel therapeutic targets to improve standard of care. Focal Adhesion Kinase (FAK) is overexpressed and phosphorylated in thyroid cancer and drives thyroid cancer growth, invasion, and metastasis. FAK is a nonreceptor tyrosine kinase that is autophosphorylated at tyrosine 397 (Y397) in response to integrin or growth factor receptor signaling, resulting in the recruitment of SRC proto-oncogene and downstream signaling pathways. FAK is predominately localized at the plasma membrane but has recently been shown to accumulate in the nucleus as well as the nucleolus to drive tumor growth. The nucleolus is a membraneless subnuclear organelle that is involved in ribosomal biogenesis through the transcription, processing, and assembly of ribosomal RNA (rRNA). The role of FAK in ribosome biogenesis is currently unknown.

**Methods:**

Nuclear/nucleolar FAK localization and function were studied using genetic and pharmacological approaches. High resolution microscopy was used to study the subcellular localization of FAK. Functional and biochemical assays including transformation and clonogenic assays, polysome profiling, and nascent protein synthesis assays were utilized to assess cell growth and survival. Protein-protein interactions of FAK were determined using a proximity dependent biotinylation (BioID) proteomics approach.

**Results:**

We have found that pY397 FAK accumulates in the nucleolus of advanced thyroid cancer cells and that autophosphorylation of FAK at pY397 and FAK kinase activity are important for nucleolar accumulation of FAK. Furthermore, knockdown of nucleophosmin 1 (NPM1), an important structural component of the nucleolus, reduced pY397 FAK nucleolar accumulation. Functionally, we showed that nuclear FAK and FAK kinase activity are necessary for anchorage independent growth. We demonstrated that targeted degradation of FAK results in decreased protein synthesis with a specific decrease in the 60S ribosomal subunit. Using a BioID proteomics approach, we showed that autophosphorylated FAK interacts with a network of nucleolar proteins including nucleolar protein 56 (NOP56) which is a core small ribonucleoprotein (snoRNP) important for 60S ribosome biogenesis. Finally, we found that pY397 FAK co-localizes with NOP56 and that knockdown of NOP56 phenocopies FAK depletion.

**Conclusions:**

Overall, these findings highlight a novel function for FAK in promoting ribosome biogenesis and suggest that nucleolar FAK represents a promising therapeutic target.

## Introduction

Thyroid cancer is the most common endocrine malignancy with 43,800 new cases reported in the United States in 2022 (1). Patients with unresectable, advanced thyroid cancer have only a 10-year survival of approximately 40-42% (2). Therefore, there is a critical need to identify novel therapeutic targets in the treatment of thyroid cancer as well as biomarkers to determine who will develop more advanced disease.

Focal Adhesion Kinase (FAK) is a promising therapeutic target that is overexpressed and phosphorylated in a variety of cancers including thyroid cancer to drive growth, survival, migration, and metastasis (3–13). FAK is a nonreceptor tyrosine kinase that interacts with growth factor receptors and integrins to promote the autophosphorylation of tyrosine 397 (pY397) FAK which recruits SRC proto-oncogene (SRC) and leads to the activation of downstream signaling pathways (14). FAK possesses a nuclear localization sequence (NLS) and has been shown to accumulate in the nucleus to drive survival, regulate inflammation, and promote immune evasion (15–17). Nuclear pY397 FAK has been correlated with decreased survival and poor prognosis in colorectal cancer and in breast cancer (18, 19). Interestingly, pY397 FAK has recently been shown to accumulate in the nucleolus in breast cancer to regulate cell growth through the nucleolar protein, nucleostemin (20). However, it remains to be determined whether FAK regulates ribosomal biogenesis which is the primary function of the nucleolus.

The nucleolus is the largest membrane-less organelle in the nucleus and is responsible for the transcription, processing, and assembly of ribosomal RNA (rRNA) to synthesize ribosomes (21). Cancer cells possess an increased number and size of nucleoli to promote rRNA transcription and protein synthesis, which are necessary to support the increased demands of tumor growth. The eukaryotic ribosome is comprised of the small subunit (40S) and large subunit (60S). The 40S subunit is formed by the 18S rRNA and 33 ribosomal proteins while the 60S subunit is formed by the 5S, 5.8S, and 28S rRNAs and 46 ribosomal proteins (22). The nucleolus is highly structured and is composed of three regions: the fibrillarin center (FC), the dense fibrillarin compartment (DFC), and the granular component (GC) (23). In the FC, ribosomal DNA (rDNA) is transcribed by RNA Polymerase I (POLI) to produce a long primary transcript, 47S pre-rRNA (21). In the DFC, the primary transcript undergoes processing and cleavage of the internal transcribed spacer 1 and 2 (ITS1 and ITS2) and 5’ and 3’ external transcribed spacers (5’- ETS1 and 3’-ETS2) to form the 18S, 5.8S, and 28S rRNAs (21, 22). Furthermore, core box c/d small ribonucleoproteins (snoRNPs), including fibrillarin (FBL), nucleolar protein 56 (NOP56), and nucleolar protein 58 (NOP58), promote the 2’-O-methylation of pre-rRNAs to influence assembly and function of the 60S ribosome (24). In the GC, rRNA in combination with ribosomal proteins forms pre-ribosomal subunits to be exported to the cytoplasm.

This study defines the localization and function of FAK in the nucleolus in advanced thyroid cancer. First, we identified that nuclear and phosphorylated FAK are required for anchorage independent growth. We discovered that pY397 FAK is localized to the nucleolus in thyroid cancer cells and that phosphorylation of Y397 FAK and NPM1 are important for FAK nucleolar accumulation. We found that targeted degradation of FAK decreases clonogenic growth as well as decreases global protein synthesis, actively translating ribosomes, and 60S large ribosomal subunits. Furthermore, we identified that pY397 FAK co-localizes with NOP56, a core snoRNP involved in 60S ribosome biogenesis, as a potential mediator to regulate clonogenic growth. Together, our data demonstrate that nucleolar FAK promotes ribosome biogenesis and protein synthesis to drive thyroid cancer growth and survival, and thus presents a promising therapeutic target to treat thyroid and other FAK-driven cancers.

## Materials and methods

### Cell lines and culture conditions

All cell lines were authenticated by Short Tandem Repeat DNA profiling using the Applied Biosystems Identifier kit (#4322288) or Globalfiler^®^ System (#4476135) at the University of Colorado Barbara Davis Center (BDC) Bioresources Core and tested for mycoplasma using the Mycoalert system (Lonza Bioscience). STR genotypes were compared to previously published data by our lab (25, 26). Cells were not used past passage 20. BCPAP (RRID: CVCL_0153) cells were kindly provided by Dr. M. Santoro, respectively, in 2007. Dr. J. Fagin kindly provided 8505C (RRID: CVCL_1054) cell lines in 2007. KTC1 (RRID: CVCL6300) and KTC2 (RRID: CVCL_6476) cells were generously provided by Dr. Kurebayashi in 2008. HCT116 TP53 isogenic cell lines including HCT116 TP53 (+/+), HCT116 TP53 (-/-), HCT116 TP53 (R248W/+), and HCT116 TP53 (R248W/-) (RRID: CVCL_0291, CVCL_HD97, CVCL_RJ69, and CVCL_RJ68 respectively) were generously provided by Dr. N. Papadopoulas in 2024 through the Johns Hopkins Biorepository & Cell Center. Human thyroid cancer cells (BCPAP, 8505C, KTC1, KTC2, C643 (RRID: CVCL_5969), ACT1 (RRID: CVCL_6291), CUTC5 (RRID: CVCL_W916), and CUTC60 (RRID: CVCL_VM61) were grown in RPMI1640 (Invitrogen) with 5% Fetal Bovine Serum (FBS). HEK293T cells (RRID: CVCL_0063) were grown in DMEM (Invitrogen) with 10% FBS (Invitrogen). Human colorectal HCT116 TP53 isogenic cell lines were grown in McCoy’s 5A Medium (Invitrogen) with 10% FBS (Invitrogen). All cells were grown in 5% CO_2_ at 37° C. KTC1 and CUTC5 were derived from pleural effusions of PTC patients. BCPAP cells were derived from a lymph node metastasis from a PTC patient. 8505C, KTC2, and CUTC60 were derived from ATC human tissue.

### Generation of FAK CRISPR/Cas9 cell lines

FAK (*PTK2*) was knocked out in the BCPAP cells using the Horizon Discovery system (Cambridge, UK). Small guide RNAs (sgRNAs) were designed using the MIT CRISPR selection tool (http://crispr.mit.edu/) and are listed in **Supplementary Table 1**. Cells were transfected with pD1301-AD:156638, which expresses Cas9, GFP, and the indicated sgRNAs targeting FAK, using Turbofect (Invitrogen, R0531) according to manufacturer’s directions. Transfection efficiency was measured by GFP visualization. 72 hours after transfection, genomic DNA was harvested using a Quick guide DNA miniprep kit (ZYMO, D3025), and mutations were screened using the SURVEYOR Mutation Detection assay (IDT, 706025). FAK-KO was confirmed by SANGER sequencing, Western blot, and qPCR analysis. The gRNA sequence used in this study is 5’-ATAATACTGGCCCAGGTGGT-3’. The identity was confirmed by STR genotyping, as above.

### pLentiV5-FAK cloning

pBabe-puro-FAK constructs expressing WT-avian-FAK, the Y397F and K454R mutants were generously provided by Dr. Filippo G. Giancotti (27). WT-, Y397F-, or K454R-FAK sequences were amplified by PCR and cloned into pLenti6/V5-D-TOPO according to manufacturer’s directions (Invitrogen, K4955). The primers are listed in **Supplementary Table 1**. The 3’ PCR primer was designed to correct a missing G at 3’ end. The FAK-nuclear localization mutant (NLM) in which the R177A, R178A, K190A, K191A, K216A, K218A sites were mutated and cloned into pLenti6/V5-D-TOPO, as above. Sequences were confirmed by SANGER sequencing in the University of Colorado BDC core. Lentivirus were packaged using Effectene Transfection Reagent (Qiagen) according to the manufacturer’s instructions. FAK-KO cells were transduced with lentivirus in presence of 8μg/mL of polybrene and selected with 10μg/mL blasticidin. Stable cell lines were validated by STR genotyping, as above.

### Lentiviral transduction and knockdowns using mission shRNA

Lentiviruses were packaged using Effectene Transfection Reagent (Qiagen) according to the manufacturer’s instructions. Cells were transduced with lentivirus in presence of 8μg/mL of polybrene and selected with 2mg/ml puromycin for 8505C or 1μg/ml puromycin for KTC2 cells. The shRNA TRC IDs are listed in **Supplementary Table 2**. Confirmation of lentiviral transductions and knockdowns were validated by immunoblotting to indicate presence of transgene or absence of protein respectively.

### Reagents

FAK PROTAC I (CAS 2301916-69-6) was obtained from MedChem Express. All antibodies used for immunoblotting and immunofluorescence are listed in **Supplementary Table 3**.

### Immunoblotting

Total protein was harvested in CHAPS lysis buffer (10 mM CHAPS, 50 mM Tris (pH 8.0), 150 nM NaCl, and 2 mM EDTA) with phosphatase and protease inhibitor cocktail (Roche). Protein was separated on an SDS polyacrylamide gel electrophoresis and transferred to Immobilon-FL polyvinylidene fluoride (PVDF) membrane (Millipore). Primary antibody incubation was performed overnight at 4°C with indicated antibodies in 5% bovine serum albumin (BSA) in TBST. Antibodies used in western blotting include pY397 FAK (Invitrogen), total FAK (BD Biosciences), NPM1 (Abcam), NOP56 (Abcam), TAMRA (Invitrogen), p53 (Cell Signaling), pY402 PYK2 (Invitrogen), total PYK2 (Cell Signaling), α-tubulin (Sigma), and vinculin (Cell Signaling). Secondary antibodies of goat anti-rabbit or goat anti-mouse IRDye (LI-COR) were applied for one hour at room temperature. Blots were imaged using the LI-COR Odyssey Imaging System and ImageStudiov4.1 software. Immunoblotting was performed in at least 2 independent biological replicates for each experiment. All antibodies used for immunoblotting are listed in **Supplementary Table 3**.

### Immunofluorescence

15,000-40,000 cells were grown on 8-well chamber slides for 48 hours prior to fixation with 4% paraformaldehyde (PFA) (Invitrogen) for 15 minutes, permeabilization with 0.5% Triton-X (Sigma) for 10 minutes, blocking with 5% normal goat serum (NGS) (Invitrogen), and incubation with primary antibodies against V5 (Cell Signaling), HA (Cell Signaling), Total FAK (BD Biosciences), pY397 FAK (Invitrogen), and fibrillarin (Invitrogen) overnight at 4°C. Slides were then incubated for one hour with Alexa Fluorophores 488 Goat Anti-Rabbit (Invitrogen) at 1:500 and Alexa Fluor 555 Goat Anti-Mouse (Invitrogen) at 1:500 dilution followed by staining with DAPI (3 μg/mL) (Invitrogen) for 30 minutes. Chambers were then removed, and samples were cured with Prolong Diamond Anti-Fade Mounting Media (Invitrogen). Images were acquired using the FV1000 Olympus Confocal Microscope with 60X or 100X objectives at the University of Colorado Advanced Light Microscopy Core (ALMC). For high resolution microscopy, thyroid cancer cells were plated on 1.5H coverslips (MatTek), and fixed, permeabilized, and blocked as stated above. Secondary antibodies of Star Red and Star Orange (Abberior) were utilized at 1:200 dilution. Images were acquired on STED Abberior Stedycon Instrument in the ALMC. In all experiments, at least 30 cells were imaged from at least two independent biological replicates. All antibodies used for immunofluorescence are listed in **Supplementary Table 3**.

### Structural modeling of FAK

A homology model of human FAK kinase and FERM domain was constructed from the human FAK sequence using the avian (*gallus gallus)* Protein Data Bank of [2J0J] and [6CB0], using the Prime module of Schrodinger release 2022-4 (19, 28–31). The resulting model was subjected to an energy minimization, referred to as the “parent” homology model. The parent homology model was separately altered with mutations in the nuclear localization sequence of FAK (comprising of R177A/R178A, K190A/K191A, K204A/K205A, and K216A/K218A), referred to as the “NLM FAK mutant”. The NLM FAK mutant was subjected to energy minimizations and overlaid onto the parent model to evaluate secondary structural changes that arise from the mutations. Structures, domains, regions, and residues of interest were labelled (32, 33).

### Proximity-dependent biotin identification mass spectrometry

WT FAK and Y397F FAK were cloned to the C-terminus of BioID2 in myc-BioID2-MCS (kindly provided by Kyle Roux; RRID: Addgene plasmid #74223). FAK was amplified using primers listed in **Supplementary Table 1** and cloned using Gibson Assembly (NEB E5510S) into the BioID2 vector cut with EcoRI and BamHI. SANGER sequencing was performed to verify sequence identity. Lentiviruses were packaged using Effectene Transfection Reagent (Qiagen) according to the manufacturer’s instructions, and virus was transduced into BCPAP thyroid cancer cells. Cells were selected with 0.5mg/ml G418 to create stable clones. 4.0 x 10^6^ BCPAP cells were plated and treated with 16 hours of 50 mM of EZ-Link Biotin (Invitrogen) prior to harvesting in BioID lysis buffer (50mM Tris, 500mM NaCl, 0.2% SDS, 1mM DTT, and 1X protease/phosphatase inhibitor). Lysates were pulled down with streptavidin-magnetic beads (Dynabeads MyOne Streptavidin T1, Invitrogen). Mass Spectrometry was performed in the University of Colorado Cancer Center Mass Spectrometry Proteomics Shared Resource (RRID: SCR_021988) using the Qexactive HF Orbitrap coupled to the nanoEasy 1000 chromatography system as described (34). Two independent experiments were performed. Data analysis included spectral processing and identification using the Mascot search program. Subtraction of proteins identified in the vector control samples along with CRAPome database was used to identify common nonspecific interactors. Data analysis included spectral processing and subtraction of proteins identified in the vector control samples with CRAPome database to identify common nonspecific interactors. Gene Ontology and String Analysis were performed on proteins that were identified in both datasets with a spectral count greater than two-fold of the WT versus EV.

### RNA isolation and quantitative RT-PCR

1.2x10^6^ KTC1 cells and 1.0x10^6^ KTC2 cells were plated in 10 cm dishes and treated with 0, 4, 8, or 16 hours of FAK PROTAC. RNA was isolated with the RNeasy Mini Kit followed by on‐column DNase digest (Qiagen). cDNA was synthesized using the High-Capacity c-DNA Reverse Transcription kit (ABI-P/N 4368814). Real time PCR reactions were carried out in MicroAmp optical 96-well reaction plates (PE ABI-N8010560) in a 20 μl mix containing 1X-Power SYBR™ Green PCR master mix (Life Technologies 4367659), 250 nM forward primer, 250 nM reverse primer and 5 μl template cDNA. The mRNA for 28S, 18S, and 5.8S were measured by real-time quantitative RT-PCR using ABI QuantStudio 7 flex Sequence detector in the University of Colorado PCR core. Thermal cycling conditions were as follows: Initiation was performed at 50°C for 2 min followed by activation of TaqGold at 95°C for 10 min. Subsequently 40 cycles of amplification were performed at 95°C for 30 secs followed by 60°C for 30 secs and 72°C for 30 secs. Melt curve analysis was performed to confirm the specificity of the amplicons for each target. Quantities of targets in test samples were normalized to the corresponding h*GAPDH* (Life Technologies-4448489). All primer sequences are provided in **Supplementary Table 1**. Three independent biological replicates were performed for each experiment.

### Polysome profiling

3 x 10^6^ BCPAP, 1.5 x 10^6^ 8505C, 3 x 10^6^ KTC1, or 2.5 x 10^6^ KTC2 cells were plated in 6 x 15 cm dishes and treated with DMSO or 1 μM FAK PROTAC for 24 or 48 hours. Cell confluency was approximately 70% at the time of harvest. The culture media was supplemented with 100 μg/mL cycloheximide (CHX) (Sigma) for 10 minutes prior to lysis with polysome preparation lysis buffer (20 mM HEPES pH 7.4, 15 mM MgCl_2_, 200 mM NaCl, 1% Triton X-100, 100 μg/ml CHX, 2 mM DTT, and 100 U RNasin) to halt translation. Supernatants were collected after centrifugation at 16,000 x *g*, 4°C for 15 minutes and 400 μl were loaded on 10–60% sucrose gradients in SW41 tubes in lysis buffer lacking Triton-X-100. Samples were ultracentrifuged at 160,000 × *g* for 3 hours at 4 °C and then fractionated using a BioComp system at 4°C, monitoring absorbance at 260 nm. Three independent biological replicates were performed in KTC2 cells and at least two replicates were performed in BCPAP, 8505C, and KTC1 cells.

### Nascent protein synthesis assays

1.2 x 10^6^ BCPAP, 0.8 x 10^6^ 8505C, 1.2 x 10^6^ KTC1, 1.0 x 10^6^ KTC2 cells, and 2.5 x 10^6^ HCT116 TP53 isogenic cell lines were plated in 10 cm dishes and treated with DMSO or 1 μM FAK PROTAC for 24, 48, or 72 hours. At the end of the time point, cells were washed twice with PBS and incubated with methionine-free RPMI media (Invitrogen) for one hour. Cells were pre-treated with water or 10 μg/ul of Cyclohexamide (CHX) (Sigma) for 30 minutes prior to a one-hour incubation with 50 μM of L-Homopropargylglycine (HPG) (Invitrogen). Lysates were incubated with 4 mM of tetramethylrhodamine (TAMRA) using the Protein Synthesis Click-it Kit (Invitrogen). Immunoblotting of lysates with antibodies against TAMRA and vinculin were utilized to assess nascent protein synthesis and loading. Three independent biological experiments were performed for each experiment.

### Colony formation assay

100–1000 cells were seeded in 6-well plates. Cell medium was changed every 3–4 days for 10–14 days. Colonies were fixed (10% methanol/10% acetic acid) and stained with 0.4% crystal violet. Crystal violet was dissolved in fixative and absorbance was measured at 570 nm using Odyssey CLx imager (Image Studio Acquisition Software Version 5.2.5, LICOR). Three independent biological replicates were performed for each experiment.

### Statistical consideration

Statistical analyses and P value calculations were performed using Graphpad Prism v8. Quantitative data are expressed as mean +/- standard error of the mean unless otherwise noted. Analysis of variance (ANOVA) was used to identify significant differences in multiple comparisons. Paired *t*-tests were used to compare two groups. All experiments were completed in at least two independent experiments with statistical tests calculated on at least 3 independent biological replicates. For all statistical analyses, the level of significance was set at 0.05. Unless otherwise noted, ns=not significant, * = p < 0.05, ** = p < 0.01, *** = p < 0.001, **** = p < 0.0001.

## Results

### Nuclear FAK and FAK kinase activity are required for anchorage independent growth

**Figure 1:**
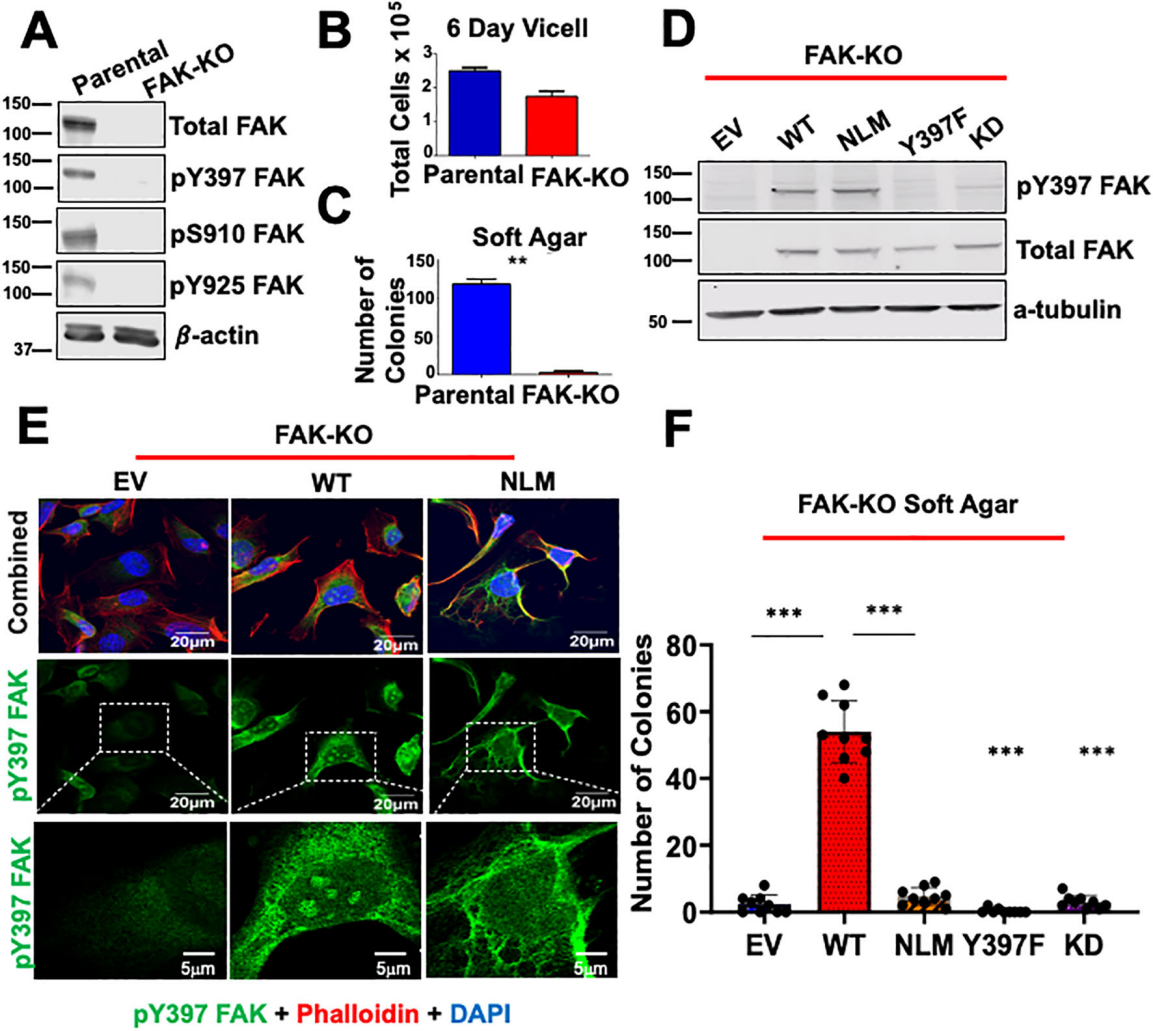
Excluding FAK from the nucleus decreases anchorage independent growth. **(A)** Parental and FAK Knockout CRISPR cells (FAK-KO) were immunoblotted with antibodies against pY397 FAK, pS910, pY925, total FAK, and β-actin. Molecular weight (MW) marker is shown on the left of each blot in kiloDaltons (kDa). **(B)** Parental and FAK-KO cells were plated for a 6 Day Vi-cell and total number of cells per mL was recorded. **(C)** Soft agar transformation assays were performed for parental and FAK-KO cells with the number of colonies counted after three weeks. **(D)** FAK-KO cells transduced with EV, WT FAK, NLM FAK, Y397F FAK, and KD FAK were immunoblotted with antibodies against pY397 FAK, total FAK, and α-tubulin. MW marker is shown on the left of each blot in kDa. **(E)** FAK-KO cells transduced with EV, WT FAK, and NLM FAK were stained with antibodies against pY397 FAK, Phalloidin, and DAPI prior to imaging at 60X with scale bar at 20μm. Inserts of zoomed in images have a scale bar of 5μm. At least 50 cells were imaged in three independent experiments. **(F)** FAK-KO CRISPR cells transduced with EV, WT FAK, NLM FAK, Y397F, and K454R were grown in soft agar assays, and the number of colonies were counted after three weeks. Experiments were performed in biological triplicates. Results are displayed as mean +/- SEM. **p < 0.01; ***p < 0.001.

Given that nuclear pY397 FAK has been correlated with poor survival in colorectal and breast cancer, we aimed to determine the role of nuclear FAK in thyroid cancer. To specifically study nuclear FAK in cells devoid of endogenous FAK, we generated a FAK knockout (FAK-KO) model in the *BRAF*-mutant BCPAP thyroid cancer cell line using CRISPR/Cas9. As expected, these cells successfully knocked out FAK protein (p<0.0001) and well-characterized phosphorylation sites, including the Y397 autophosphorylation site, the Src-dependent Y925 site, and S910, which is a MAPK-dependent site (**Figure 1A**; **Supplementary Figure 1A** **).** Consistent with our previously published data using shRNA to knockdown FAK, FAK-KO decreased thyroid cancer adherent growth by 55% (**Figure 1B**, not significant) and nearly ablated anchorage independent growth by 25-fold (p < 0.01) (35) (**Figure 1C**). To determine the specific role of nuclear FAK in growth and survival, we rescued the FAK-KO cells by transducing with empty vector (EV), wild type FAK (WT), a nuclear localization mutant of FAK to exclude FAK from the nucleus (NLM), in which the NLS was mutated, a non-phosphorylatable FAK mutant (Y397F), and a kinase dead FAK (KD). We confirmed similar levels of WT FAK expression among the rescues and confirmed significantly decreased pY397 FAK expression levels in the Y397F FAK and KD FAK rescues compared to the WT (p<0.05) (**Figure 1D**; **Supplementary Figure 1B**). Finally, we confirmed that the FAK-NLM successfully excluded pY397 FAK from the nucleus by immunofluorescence (**Figure 1E**). To determine the functional role of nuclear FAK, we performed soft agar transformation assays and found that WT FAK is sufficient to rescue soft agar in the FAK-KO cells. However, excluding FAK from the nucleus (FAK-NLM), expression of a Y397F autophosphorylation mutant, or kinase dead FAK, failed to rescue soft agar (p<0.001) (**Figure 1F**). Together, these data indicate that nuclear FAK, along with FAK autophosphorylation and kinase activity are necessary for anchorage independent growth, which is phenocopied by loss of FAK autophosphorylation and kinase activity.

In **Figure 1**, we observed that pY397 FAK exhibited punctate nuclear staining in the FAK-WT rescue cells but not in the nuclear-localization mutant (FAK-NLM) cells. NLS sequences can serve as nuclear as well as nucleolar localization sequences for proteins (36–38). To further delineate the importance of the NLS on FAK structure, we performed structural modeling of the avian-based FAK model. We found that modeling global mutations in the NLS (R177/178A, K190/191A, K204/205A, and K216/218A) promote β-sheet disordering around the regions of 399–402 compared with the parental structure (**Supplementary Figure 2**). Thus, these data suggest that the NLS of FAK is important for the structure around the pY397 FAK residue, which could regulate nuclear and/or nucleolar accumulation.

### pY397 FAK is present in the nucleolus of thyroid cancer cells and is important for nucleolar accumulation of FAK

**Figure 2:**
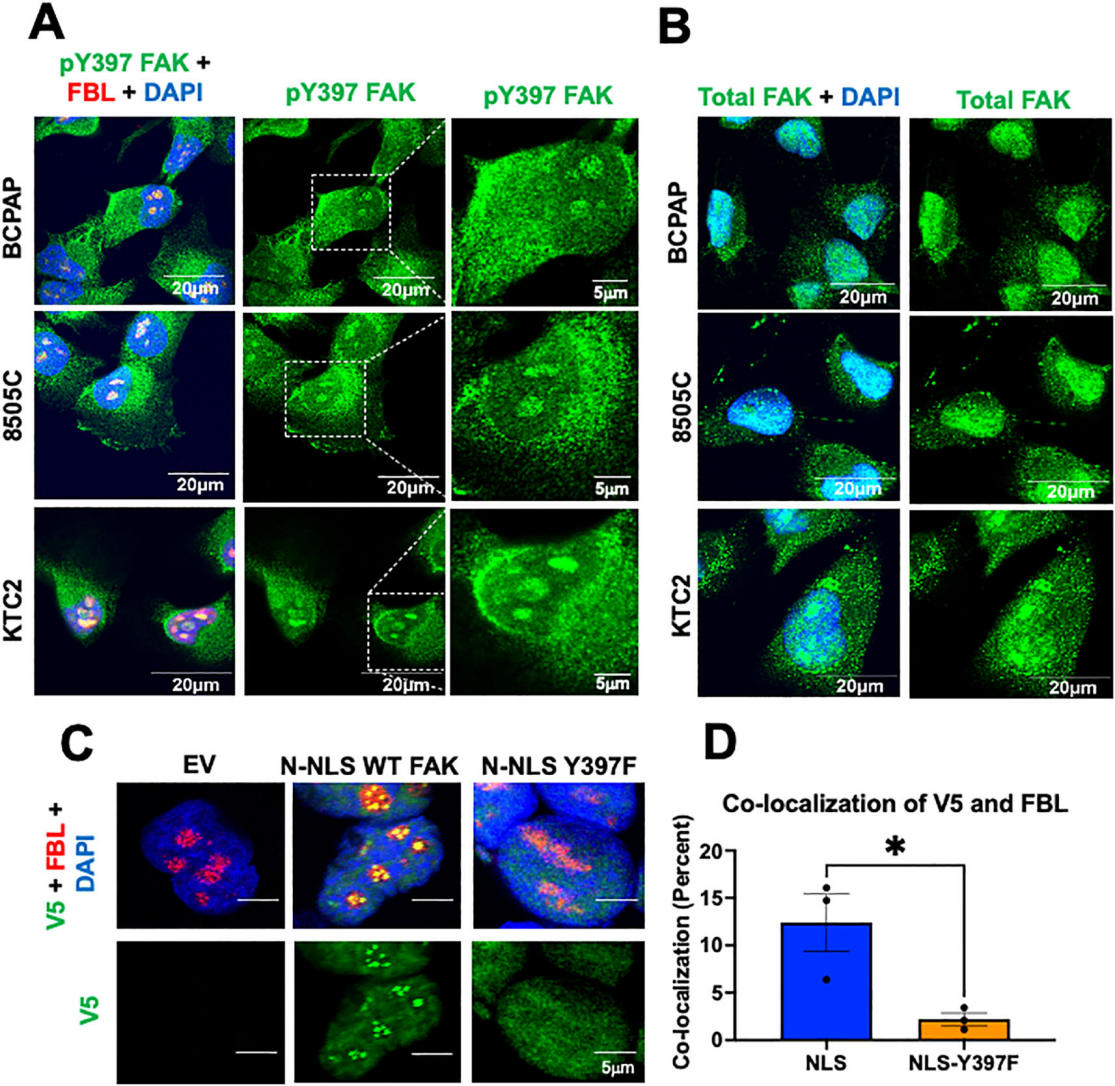
pY397 FAK localizes to the nucleolus and influences nucleolar FAK accumulation **(A)** BCPAP, 8505C, and KTC2 cells were stained with antibodies against pY397 FAK, FBL, and DAPI prior to confocal imaging at 100X. **(B)** BCPAP, 8505C cells, and KTC2 cells were probed with antibodies against total FAK and DAPI prior to imaging at 100X confocal. Results shown are representative images from 20–30 images taken in at least two independent experiments. Scale bar represents 20μM. **(C)** FAK-KO cells transduced with EV, N-terminal NLS FAK, and N-terminal NLS-Y397F were probed with antibodies against V5, Fibrillarin, and DAPI prior to imaging at 100X on Olympus Confocal. At least 50 cells were imaged in three independent experiments. Scale bars represent 5μm. **(D)** Percent of co-localization between V5 and FBL in NLS versus NLS-Y397F KO-FAK transduced cells using Integrated Density Analysis by Image J. Results displayed as mean +/- SEM. *, p<0.05.

Given the subnuclear punctate accumulation of pY397 FAK, we next aimed to investigate the accumulation of pY397 FAK in a panel of thyroid cancer cells with BRAF V600E and RAS mutations. BRAF V600E mutations occur in approximately 50-70% of PTC, and mutations in RAS occur in approximately 40-50% in FTC (39). We found that a fraction of pY397 FAK co-localizes with the nucleolar marker, FBL, in the panel of *BRAF-* and *RAS-*mutant thyroid cancer cell lines derived from PTC or ATC tumors (**Figure 2A**; **Supplementary Figure 3**). We next evaluated the accumulation of total FAK in thyroid cancer cell lines expressing BRAF V600E mutations due to their association with recurrence, extrathyroidal extension, advanced stage, and distant metastases in thyroid cancer (26, 39). While we found pY397 FAK specifically localizes in the nucleolus, we found that total FAK is distributed throughout the nucleus (**Figure 2B**). Thus, these data suggest that pY397-FAK is enriched in the nucleolus in advanced thyroid cancer cells, while total FAK is localized throughout the nucleus, as previously observed (15, 17) suggesting that pY397-FAK plays a specific role in the nucleolus.

The phosphorylation of proteins has been shown to facilitate nucleolar accumulation (40, 41). Therefore, we asked if pY397 FAK is important for nucleolar accumulation of FAK. Given that NLS sequences can serve as nuclear as well as nucleolar localization sequences for proteins, we fused 3 NLS sequences (PKKKRKV) from an SV40 NLS onto the N-terminus of WT FAK (NLS-FAK) or a non-phosphorylatable FAK mutant (NLS-Y397F-FAK) plasmid which possess a V5 tag and transduced these constructs into the FAK-KO CRISPR BCPAP cell line. We confirmed that these constructs are sufficient to induce nuclear FAK accumulation (**Supplementary Figure 4A** **).** Notably, we found that expression of NLS-WT-FAK results in discrete punctate localization of FAK that specifically co-localizes with the nucleolar marker, fibrillarin (FBL) (**Figure 2C**). Thus, 3x NLS is sufficient to induce nucleolar FAK accumulation. Next, we targeted FAK into the nucleus with a non-phosphorylatable Y397F-FAK mutant. Interestingly, we found that the NLS-Y397F-FAK resulted in significantly decreased nucleolar FAK accumulation (3- fold decrease) (p<0.05) (**Figure 2C, D** **),** indicating that pY397 FAK is important for nucleolar accumulation. To corroborate our results, we utilized an additional model with NLS fused to the C-terminus of the WT FAK or NLS-Y397 FAK and similarly found that targeting FAK to the nucleus increases nucleolar FAK which is reduced in the NLS-Y397F-FAK (**Supplementary Figure 4B** **).** Overall, these data indicate that pY397 is localized in the nucleolus of advanced thyroid cancer cells and pY397 FAK is important for FAK nucleolar accumulation.

### NPM1, a key protein of the granular component, contributes to pY397 FAK accumulation in the nucleolus

**Figure 3:**
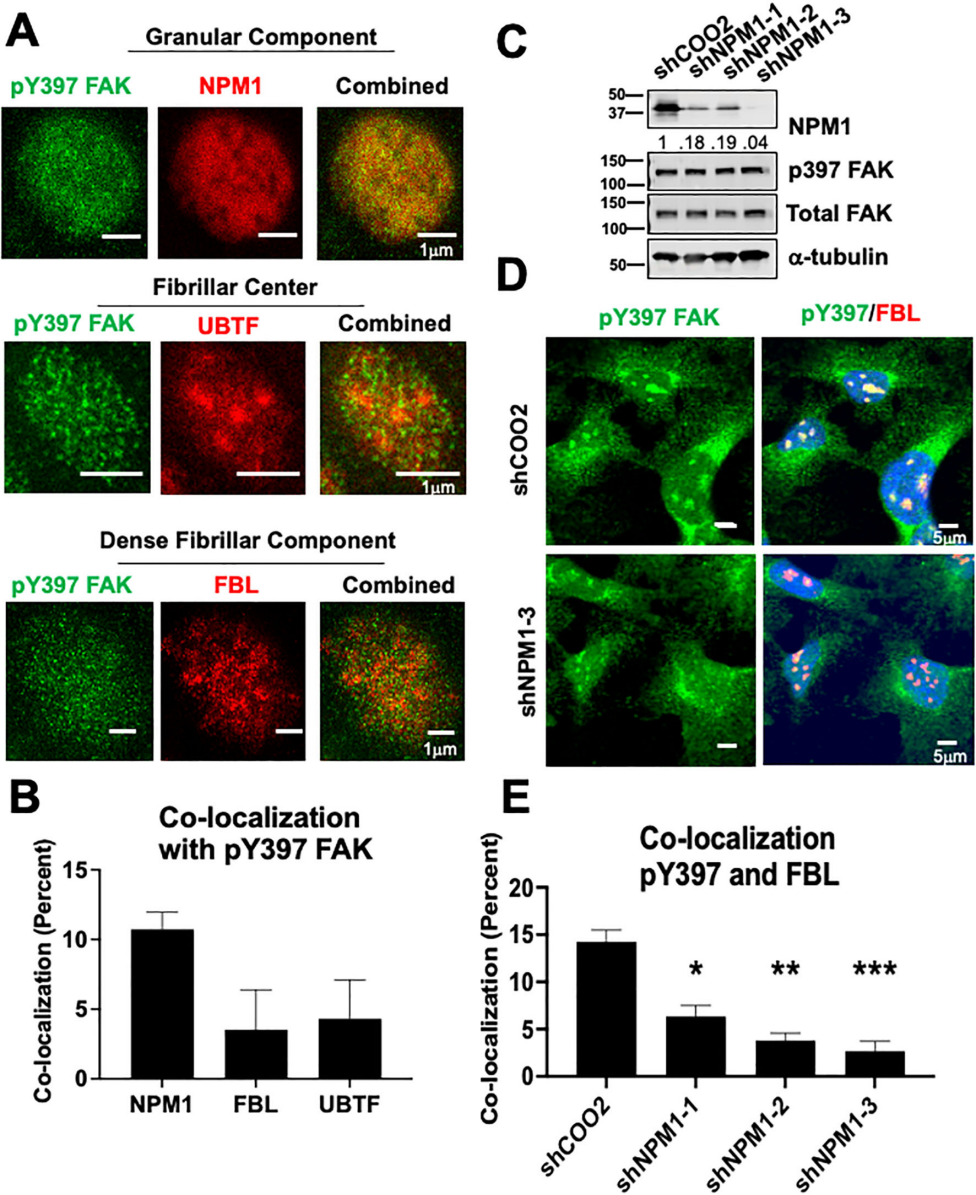
NPM1 is necessary for pY397 FAK localization to the nucleolus in adherent cells **(A)** BCPAP cells were probed with antibodies against NPM1, FBL, and UBTF and imaged using STED microscopy. At least 50 cells were visualized in three independent experiments. Scale bar represent 1μm. **(B)** Percent of co-localization between pY397 FAK and NPM1, UBTF, and FBL respectively using Integrated Density Analysis by Image J.**(C)** 8505C cells were knocked down with either a non-targeting control or three shRNAs targeting NPM1 prior to immunoblotting with antibodies against NPM1, pY397 FAK, total FAK, or α-tubulin. Quantification of NPM1 expression is normalized to α-tubulin and sh-COO2 control and is listed under the NPM1 blot. MW marker is shown on the left of each blot in kDa. Two independent experiments were performed. **(D)** Immunofluorescence was performed in 8505C shCOO2 or shNPM1 cells probed with antibodies against pY397 FAK, FBL, and DAPI. Cells were imaged at 60X confocal imaging in three independent experiments with visualization of at least 50 cells. Scale bar represent 5 μm. **(E)** Percent of co-localization between pY397 FAK and FBL in our non-targeting controls and shNPM1 knockdown cells using Integrated Density Analysis by Image J. Results displayed as mean +/- SEM. *, p<0.05; **p < 0.01; ***p < 0.001.

The nucleolus is a highly compartmentalized organelle with each layer representing a different nucleolar function. To better define the localization and subsequent function of nucleolar pY397 FAK, we performed high resolution 2D stimulated emission depletion (STED) microscopy with antibodies against nucleophosmin 1 (NPM1), fibrillarin (FBL), and upstream binding transcription factor (UBTF) as markers for the granular component, dense fibrillarin component, and fibrillarin center respectively. We found that pY397 FAK accumulates in all three nucleolar layers and is potentially enriched in the granular component, with a >10% co-localization with NPM1 in this representative cell line (**Figure 3A, B**, 2.5-fold enrichment, not statistically significant**).** pY397 FAK also co-localizes with FBL (fibrillarin center marker) and UBTF (dense fibrillarin layer marker) at <5% (**Figure 3A, B** **).** Given the potential enrichment of pY397 FAK in the granular component, we focused on the role of FAK in the granular component. To assess if the granular component is necessary for FAK accumulation, we knocked down *NPM1* with three different shRNAs and confirmed expression by western blot. The protein expression of pY397 FAK and total FAK was not affected by *NPM1* knockdown (**Figure 3C**). Upon *NPM1* knockdown, we observed a notable decrease in pY397 FAK nucleolar accumulation (**Figure 3D, E**). Thus, NPM1 which is the critical component of the granular compartment contributes to pY397 nucleolar accumulation.

### Targeted FAK degradation decreases protein synthesis, translation, and clonogenic growth

**Figure 4:**
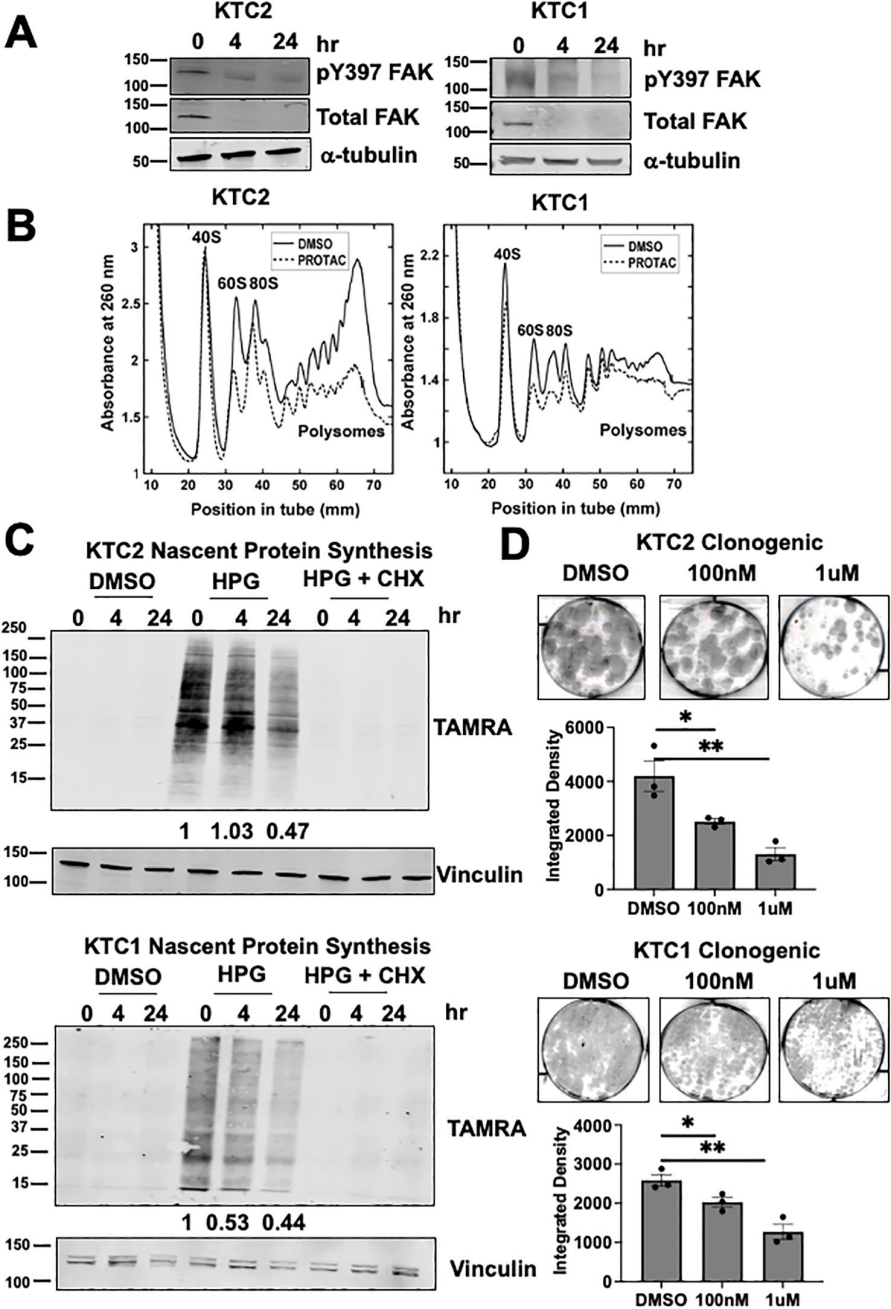
FAK regulates 60S biogenesis, nascent protein synthesis, and thyroid cancer growth **(A)** KTC1 and KTC2 cells were treated with 0, 4, or 24 hours of 1 μM PROTAC and immunoblotted with antibodies against pY397 FAK, total FAK, or α-tubulin. MW marker is shown on the left of each blot in kDa. **(B)** Polysome profiling of KTC1 and KTC2 cells treated with DMSO or 1 μM PROTAC for 24 hours was performed with absorbance measured at 260nm. Polysome profiling of KTC2 cells were performed in biological triplicate and KTC1 cells were performed in two biological replicates. **(C)** KTC1 and KTC2 cells were treated with DMSO or 1 μM PROTAC for 24 hours prior to methionine starvation, pre-treatment with water or CHX, and 1 hour treatment with methionine analog HPG. Lysates were immunoblotted with antibodies against TAMRA and vinculin. MW marker is shown on the left of each blot in kDa. At least two independent replicates were performed for all cell lines. **(D)** KTC1 and KTC2 cells were grown in clonogenic assays, treated with DMSO, 100 nM, or 1μM PROTAC, and stained with crystal violet after 14 days. Quantification of clongenic growth was performed by integrated density analysis using LICOR Odyssey. Experiments were performed in biological triplicate. Results are displayed as mean +/- SEM. *, p < 0.05; **, p < 0.01.

We next evaluated the regulation of ribosome biogenesis and protein synthesis by FAK. To quickly and selectively target FAK, we used a proteolysis targeting chimera (PROTAC) approach, which utilizes the ubiquitin proteasome system, to degrade a protein of interest. Furthermore, PROTACs are emerging as a new therapeutic approach in precision medicine (42). The FAK PROTAC consists of the FAK kinase inhibitor, defactinib, fused via a linker to the von Hippel-Lindau (VHL) E3 Ligase to specifically target and degrade FAK. With targeted degradation of FAK, we found an effective decrease in total FAK protein within 4 hours of FAK PROTAC treatment and a corresponding decrease in pY397 FAK expression in 24 hours in *BRAF*-mutant, KTC1 and KTC2, thyroid cancer cells (**Figure 4A**). Residual pY397 FAK expression upon treatment with FAK PROTAC may be due to cross-reactivity of this antibody with the highly related kinase, PYK2 (*PTK2B*) (43). We also found that degradation of FAK did not significantly alter the transcription of the mature 18S, 28S, or 5.8S rRNAs which is expected given the long half-life of rRNAs of 3 to 7 days (44) (**Supplementary Figure 5A** **).** To assess if the targeted degradation of FAK impacts the association of mRNA and ribosomes, we then performed polysome profiling with sucrose gradients. At baseline, the polysome traces of the KTC2 and KTC1 cells show an imbalance in subunit abundance with a higher 40S/60S ratio, along with the presence of polysomes, indicating active translation (**Figure 4B**). Upon targeted FAK degradation, we identified a significant reduction of the 60S large ribosomal subunit (p<0.05) and loading of mRNA on polysomes (p<0.001), indicating a decrease in active translation in KTC2 cells and similar trend in KTC1 cells (**Figure 4B**; **Supplementary Figure 5B** **).** Accordingly, we found a >50% decrease in global nascent protein synthesis upon FAK degradation in the presence of the methionine analog, HPG, in KTC2 and KTC1 cells (**Figure 4C** **).** This decrease in protein synthesis is likely not due to a global decrease in cell growth, as we only observed a ~20% reduction in cell number at 24 hours (**Supplementary Figure 5C** **).** However, loss of FAK leads to a decrease in cell growth at later time points with a 40-60% inhibition of cell number at 3 and 6 days (**Supplementary Figure 5C** **).** Finally, we observed a 4-fold decrease and 2.5-fold decrease in clonogenic growth in KTC2 and KTC1 cells, respectively, upon 14 days of 1 μM FAK-PROTAC treatment compared to DMSO (p<0.01) (**Figure 4D** **).** Together, our data indicate that FAK regulates 60S ribosomal biogenesis, impacting loading of mRNA on polysomes, nascent protein synthesis, and ultimately thyroid cancer growth.

Next, we addressed the role of FAK degradation in the *BRAF*-mutant thyroid cancer cells, BCPAP and 8505C. While we identified that FAK PROTAC successfully degraded FAK within 24 hours of treatment, we found no effect on polysome profiling, nascent protein synthesis, or clonogenic growth in these cell lines (**Supplementary Figure 6** **).** Of note, KTC1 and KTC2 cells express *TP53-WT* while BCPAP and 8505C cells express mutant*-TP53* (26). We hypothesized that this difference in ribosome biogenesis may be due to *TP53*-status as *TP53* actively regulates various stages of ribosome biogenesis, and *TP53*-mutant cells promote increased ribosome biogenesis compared to *TP53*-WT cells (45, 46). However, there was no difference in nascent protein synthesis in isogenic colorectal *TP53* HCT116 cell lines upon degradation with a FAK PROTAC at 24 and 48 hours (**Supplementary Figure 7**). Thus, further investigation is warranted to investigate the underlying differences between these cell lines.

### pY397 FAK co-localizes with NOP56 as a potential mediator to regulate 60S biogenesis and thyroid cancer growth

**Figure 5:**
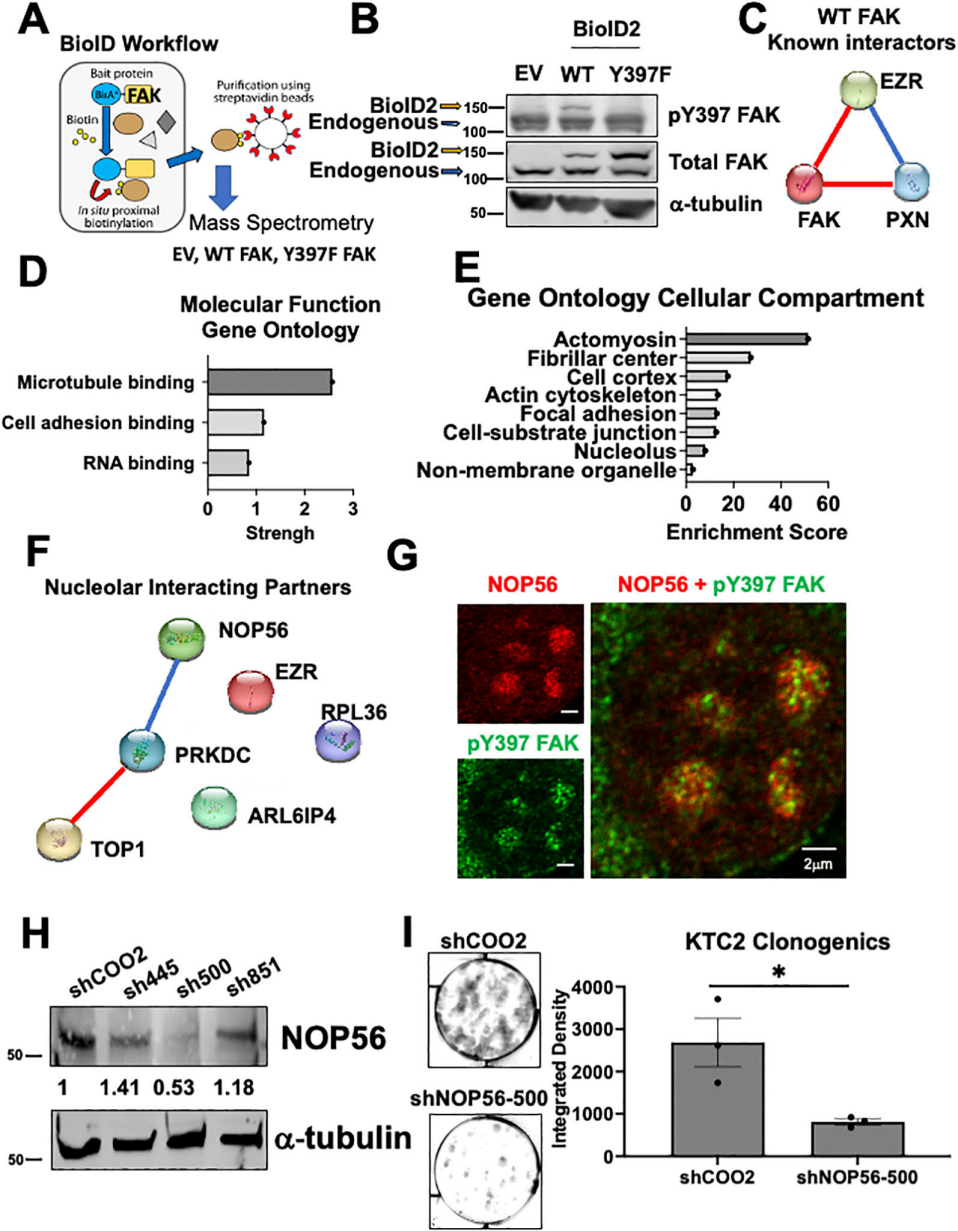
NOP56 co-localizes with pY397 FAK and promotes thyroid cancer growth **(A)** BioID workflow representing FAK fused to a promiscuous biotin ligase and interacting partners of FAK being biotinylated, pulled down, and identified by mass spectrometry.**(B)** Immunoblotting of BCPAP cells transduced with EV, WT, and Y397F FAK BioID2 and probed with antibodies against pY397 FAK, total FAK, and α-tubulin. The higher MW band represents the 27kDA BioID2 construct fused to WT or Y397 FAK and lower MW band is the endogenous FAK band. **(C)** STRING analysis of significantly enriched proteins reveals known interacting partners of FAK. Blue connections signify known interactions from database. Red connections signify experimentally determined known interactions. **(D)** FAK interacting partners were identified by analyzing proteins that bind to WT FAK in both datasets and enriched at least 2-fold change compared to EV. Gene Ontology Pathway Analysis was conducted for molecular function pathways with a false discovery rate (FDR) <0.05. **(E)** Gene Ontology Pathway Analysis was conducted for cellular compartment pathways with FDR < 0.05. **(F)** STRING analysis of nucleolar proteins identified by Gene Ontology Pathway Analysis. Blue connections signify known interactions from database. Red connections signify experimentally determined known interactions. **(G)** Confocal microscopy of KTC2 cells was performed with antibodies against pY397 FAK and NOP56. At least 50 cells were imaged at 100X with the Abberior Stedycon microscope in two independent experiments. Scale bar represent 2 μm. **(H)** Immunoblotting of KTC2 cells transduced with a non-targeting control or three NOP56 shRNAs was performed with antibodies against NOP56 and α-tubulin. MW marker is shown on the left of each blot in kDa .**(I)** Clonogenic assays of KTC2 cells transduced with non-targeting control or NOP56 shRNA were performed. Colonies were stained with crystal violet and imaged after 10 days. Integrated Density was utilized to quantify clonogenic growth using LICOR Odyssey software. Experiments were performed in biological triplicate experiments. Results are displayed as mean +/- SEM. *, p < 0.05.

To investigate how FAK regulates 60S active translation, we utilized proximity-based biotinylation mass spectrometry to identify interacting partners of FAK. Through this approach, we fused a promiscuous biotin ligase (BioID2) to wild type FAK (WT) or a non-phosphorylatable FAK mutant (Y397F) (**Figure 5A** **) (** 47). Upon addition of biotin, proteins that come within approximately 10 nm of FAK are biotinylated, pulled down, and identified by mass spectrometry. First, we confirmed expression of the 27kDa BioID2 construct fused to WT or Y397 FAK by immunoblotting (**Figure 5B** **).** To assess interacting partners of FAK, we analyzed proteins that were identified in both of our BioID biological replicates and bound WT FAK with greater than 2-fold change compared to EV. As expected, WT FAK interacts with itself as well as known interacting partners including paxillin (PXN) and ezrin (EZR) (**Figure 5C** **).** Next, we identified significantly enriched pathways that are FAK-dependent. Interestingly, we found that “RNA binding” as well as the “fibrillar center” and the “nucleolus” are significantly enriched pathways (FDR < 0.05) (**Figure 5D, E** **).** Despite the mass spectrometry being performed in whole cell lysates and the nucleolus only comprising ~7% of the entire proteome, these data indicate that regulation of nucleolar processes is a key function for pY397-FAK. The nucleolar proteins that we identified by mass spectrometry include NOP56, TOP1, PRKDC, EZR, ARL6IP4, and RBL36, all of which have functions in ribosome biogenesis (**Figure 5F** **).** Of these proteins, NOP56 passed stringent criteria to interact with WT FAK and not EV or Y397F FAK in both of our BioID replicates (**Supplementary Tables 4**–**6** **).** NOP56 is a core box C/D snoRNP that is involved in the 2’-O-methylation of pre-rRNA necessary for 28S rRNA maturation and thus 60S ribosome biogenesis. These novel findings suggest that pY397 FAK is important for 60S ribosome biogenesis and protein synthesis via NOP56. First, we confirmed that pY397 FAK and NOP56 are co-localized in the nucleolus of our thyroid cancer cells by microscopy (12% co-localization) (**Figure 5G**; **Supplementary Figure 8** **).** To confirm the functional role of NOP56, we transduced the KTC2 cells with 3 shRNAs targeting NOP56 and observed the greatest knockdown of NOP56 (approximately 50%) with shNOP56-500 (**Figure 5H** **).** Thus, we assessed the role of NOP56 on clonogenic growth using shNOP56-500. We found that knocking down NOP56 results in a 3-fold decrease in clonogenic growth compared to the non-targeting control (p<0.05) (**Figure 5I** **).** Overall, these data suggest that pY397 FAK functionally interacts with NOP56 as a potential mediator to regulate 60S biogenesis and thyroid cancer growth.

## Discussion

FAK is overexpressed and phosphorylated in a multitude of cancers including thyroid cancer and represents a promising therapeutic target to halt FAK mediated tumorigenic processes including growth, drug resistance, invasion, cell survival, and metastasis (3–13, 48–51). However, FAK inhibitors have largely been ineffective in the clinic. Therefore, it is essential to understand the biology of FAK to target FAK more efficiently and to identify the correct patient population. We have shown that pY397 FAK is localized in the nucleolus in aggressive thyroid cancer cells where it interacts with nucleolar proteins involved in ribosomal biogenesis including NOP56, TOP1, RPL36, and PRKDC. We identified that autophosphorylation of pY397 FAK is important for FAK nucleolar accumulation. We further observed a potential enrichment of pY397 FAK with NPM1 in the granular component and that NPM1 contributes to FAK nucleolar accumulation. Upon FAK degradation, we observed a decrease in the 60S large ribosomal subunit, a global reduction in actively translating ribosomes, and corresponding decrease in growth and survival. Finally, we showed that a fraction of pY397 FAK co-localizes with NOP56, a key regulator of 60S ribosome biogenesis, and that NOP56 is important for thyroid cancer growth and survival. Overall, our studies highlight that FAK regulates 60S ribosome biogenesis and global protein synthesis to drive thyroid cancer growth and survival, providing a novel therapeutic vulnerability.

In this study, we found that nuclear FAK and FAK kinase activity are necessary for anchorage independent growth using advanced thyroid cancer as a model in **Figure 1**. FAK has been shown to accumulate in the nucleus in several cancers, and elevated levels of Y397 nuclear FAK in breast cancer and colorectal cancer patient tissues are associated with a poor prognosis (18, 19). The kinase activity of nuclear FAK has also been shown to play a role in immune evasion. Specifically, nuclear FAK promotes immune evasion through increased CCL5 expression to drive exhaustion of CD8+ T cells and recruit regulatory T cells (Tregs) in the tumor microenvironment (16). While our work corroborates existing literature that nuclear FAK and FAK kinase activity promote increased thyroid cancer growth and survival, the role of nucleolar FAK in these previous studies would be of interest to evaluate.

Furthermore, our nuclear FAK model provides instrumental insight into the localization of nucleolar FAK. We found that excluding FAK from the nucleus reduces nucleolar p397 FAK accumulation in **Figure 1**. Conversely, we found that forcing FAK into the nucleus with 3x SV40 NLS results in nuclear and nucleolar accumulation in **Figure 2**. Our data suggests that a NLS (endogenous and SV40) are important for FAK nucleolar accumulation, supporting existing literature that NLS sequences can serve as nuclear as well as nucleolar localization sequences for proteins (36–38). Furthermore, we found that mutating the NLS of FAK affects the structure around pY397 FAK through structural modeling. The function of a protein may be highly influenced by its sequence, structure, and flexibility. Thus, our data highlights that the nucleolar accumulation of FAK is influenced by its NLS and that pY397 likely plays a key role in FAK nucleolar accumulation.

In further investigation of pY397 FAK, we identified that a fraction of pY397 FAK accumulates in the nucleolus of cell lines derived from advanced thyroid cancer patients and that pY397 FAK is important for FAK nucleolar localization in **Figure 2**. The nucleolus which is a membraneless organelle forms through liquid liquid phase separation (LLPS). Interestingly, phosphorylation of proteins has been shown to facilitate nucleolar accumulation. Of relevance to FAK, tyrosine phosphorylation specifically promotes liquid phase condensates through recruitment of SH domains promoting multi-valent protein-protein interactions (40, 41). Indeed, FAK has recently been shown to undergo LLPS with p130Cas (BCAR1) at the plasma membrane (52). Thus, it will be interesting to determine whether LLPS regulates FAK nucleolar accumulation in the future. Our data also supports the findings from Tancioni et al. that identified pY397 FAK accumulates in the nucleolus of breast cancer cells to drive stabilization of nucleostemin through NPM1 and AKT (20). Together, these data suggest a role for nucleolar FAK in human cancer. Overall, our data highlight that a fraction of pY397 FAK accumulates in the nucleolus of thyroid cancer cells and that pY397 FAK is important for nucleolar accumulation as well as growth and survival.

Using STED imaging, we observed that pY397 FAK may localize in all layers of the nucleolus, with a potential enrichment with NPM1 in the granular component in **Figure 3**. Using shRNA knockdown of NPM1, the main component of the granular component, we showed that NPM1 contributes to pY397 FAK nucleolar accumulation in **Figure 3**. The granular component is vital for final rRNA processing steps, rRNA assembly, and rRNA export. We identified that FAK is important for 60S subunit levels, mRNA loading, and nascent protein synthesis in **Figure 4**. Using a protein-protein interaction proteomics approach, we identified a network of nucleolar proteins that specifically interact with pY397 FAK. Of these, NOP56 plays a role in 60S ribosome biogenesis. We further showed that a fraction of pY397 FAK co-localizes with NOP56. While NOP56 is predominately found in the dense fibrillarin component, it also partitions to the granular component (53). Thus, our data offer novel insights into the role of FAK in ribosome biogenesis through influence of the 60S ribosome, mRNA loading, and nascent protein synthesis.

NOP56 is a core c/d box snoRNP that is involved in the 2’-O-methylation of pre-rRNA and assembly of the 60S ribosome (54). 2’-O-Methylation of rRNA occurs in functionally important regions of the ribosome such as the peptidyl transferase center to influence ribosome assembly and function (55). Furthermore, NOP56 is highly regulated by SUMOylation to alter its function. NOP56 has been found to be SUMOylated by USP36 which is a nucleolar deubiquitinating enzyme (56). It remains to be determined whether pY397 FAK regulates the SUMOylation and subsequent methylation activity of NOP56 in future studies.

In this study, we utilized a panel of thyroid cancer cell lines derived from advanced PTC and ATC tumors. Given the limited treatment options for ATC and unresectable, advanced PTC, identification of therapeutic targets including FAK holds substantial clinical significance (2). An important future direction includes histological evaluation of FAK localization in low risk versus high-risk PTC as well as highly aggressive ATC patient tissue.

Finally, this study offers innovative approaches to elucidate the localization and function of nucleolar FAK. We are the first group to successfully target FAK to the nucleus using 3x SV40 NLS in which we observed the importance of pY397 FAK in FAK nucleolar accumulation. This model will offer novel insight into the biochemical importance of p397 FAK in nucleolar accumulation in future studies. Of note, the NLS constructs exhibited some instability and therefore expression will need to be optimized for future experimental assays. In addition, we utilized a FAK PROTAC which is an emerging tool in precision medicine to degrade FAK (42). It enabled the efficient degradation of FAK to investigate the regulation of protein synthesis. This model provided novel insights that FAK regulates actively translating ribosomes, 60S subunit levels, and nascent protein synthesis. These findings highlight nucleolar FAK as a novel therapeutic target in cancer.

## Data Availability

The datasets presented in this study can be found in the article/**Supplementary Material**.
