# Supplementary material for: Focal adhesion kinase promotes ribosome biogenesis to drive advanced thyroid cancer cell growth and survival
Source: Front Oncol. 2025 May 19;15:1252544. doi: 10.3389/fonc.2025.1252544 (PMC12127332; doi:10.3389/fonc.2025.1252544)
Supplement: Supplementary file 1 [file Table1.pdf]

Supplementary Table 1: List of Sequences

Supplementary Table 1		
Name	Sequence (5' to 3')	Use
PTK2 gRNA	TTGGTGTGTGATTCAAGTTG	CRISPR;Cas9
PTK2 gRNA	TTCGAGTACTAAGACTCACC	CRISPR;Cas9
PTK2 gRNA	ACTGGTATGGAACGTTCTCC	CRISPR;Cas9
PTK2 gRNA	TGCAATGGAGCGAGTATTAA	CRISPR;Cas9
PTK2 gRNA	ATAATACTGGCCCAGGTGGT	CRISPR;Cas9
PTK2 for	CACCATGGCAGCAGCTTACCTTGATCC	pLenti Cloning
PTK2 rev	GTGGGGCCTGGACTGGCT	pLenti Cloning
SV40 PTK2 for	AGTCACCATGGATCCAAAAAAGAAGAGAAAGGTAGATCCAAAGAAG AAGCGAAAGGTCGATCCGAAAAAAAAAAGAAAGGTAGG	SV40 Cloning
SV40 PTK2 rev	CCTACCTTTCTTTTTTTTTTCGGATCGACCTTTCGCTTCT TCTTTGGATCTACCTTTCTCTTCTTTTTTGGATCCATGGTGACT	SV40 Cloning
PTK2 BioID for	GTGCTGGATATCTGCAGAATTCGCAGCTGCTTACCTTGACCCCAA	BioID Cloning
PTK2 BioID rev	TTGGTACCGAGCTCGGATCCTCAGTGTGGTCTCGTCTGCCCAA	BioID Cloning
28S rRNA for	AGAGGTTAAACGGGTGGGGTC	Gene Expression
28S rRNA rev	GGGGTCGGGAGGAACGG	Gene Expression
18S rRNA for	GATGGTAGTCGCCGTGCC	Gene Expression
18S rRNA rev	GCCTGCTGCCTTCCTTGG	Gene Expression
5.8S rRNA for	ACTCGGCTCGTGCGTC	Gene Expression
5.8 rRNA rev	GCGACGCTCAGACAGG	Gene Expression
Abbreviations: gRNA: guide RNA; for: forward; rev: reverse		
