# Supplementary material for: Focal adhesion kinase promotes ribosome biogenesis to drive advanced thyroid cancer cell growth and survival
Source: Front Oncol. 2025 May 19;15:1252544. doi: 10.3389/fonc.2025.1252544 (PMC12127332; doi:10.3389/fonc.2025.1252544)
Supplement: Supplementary file 2 [file Table2.pdf]

**Supplementary Table 2: shRNA TRC Clone IDs**

| Supplementary Table 2 | |
|---|---|
| **Gene shRNA** | **TRC Clone ID** |
| NPM1 | TRCN0000062268 |
| NPM1 | TRCN0000062270 |
| NPM1 | TRCN0000062272 |
| NOP56 | TRCN0000148851 |
| NOP56 | TRCN0000276500 |
