# Supplementary material for: Focal adhesion kinase promotes ribosome biogenesis to drive advanced thyroid cancer cell growth and survival
Source: Front Oncol. 2025 May 19;15:1252544. doi: 10.3389/fonc.2025.1252544 (PMC12127332; doi:10.3389/fonc.2025.1252544)
Supplement: Supplementary file 3 [file Table3.pdf]

Supplementary Table 3: List of antibodies used for Immunofluorescence and Immunoblotting

Protein	Species	Supplier/Catalog	Use	Dilution
pY397 FAK	Rb	Invitrogen #700255	IF/WB	1:1000
Total FAK	Ms	BD #610088	WB	1:1000
Total FAK	Ms	BD #610088	IF	1:50
V5	Rb	Cell Signaling #13202S	IF/WB	1:1000
NPM1	Ms	Invitrogen #32-5200	IF	1:500
NPM1	Rb	Invitrogen #FC-61991	IF	1:200
FBL	Ms	Abcam #Ab4566	IF	1:500
UBTF	Ms	Invitrogen ##H00007343-M02	IF	1:500
NOP56	Ms	Invitrogen MA5-24641	IF	1:250
p53	Rb	Cell Signaling #9282	WB	1:1000
pY402 PYK2	Rb	Invitrogen #44-618G	WB	1:1000
Total PYK2	Ms	Cell Signaling #3480S	WB	1:1000
α-tubulin	Ms	Sigma #CP06	WB	1:4000
Vinculin	Rb	Cell Signaling #13901S	WB	1:1000
NOP56	Rb	Invitrogen #MA1-041	WB	1:5000
TAMRA	Ms	Invitrogen #MA1-041	WB	1:1000
HA	Ms	Cell Signaling #2367S	IF	1:100
Alexa Fluor 488 Secondary	Goat Anti-Rb	Invitrogen #C10456	IF	1:500
Alexa Fluor 555 Secondary	Goat Anti-Ms	Invitrogen #A27039	IF	1:500
Star Orange Secondary	Goat Anti-Ms	Abberior #STORANGE-1001	IF	1:200
Star Red Secondary	Goat Anti-Rb	Abberior #STRED-1002	IF	1:200

Abbreviations: Ms: Mouse; Rb: Rabbit; IF: Immunofluorescence; WB: Western Blot
